# The elucidation of the multimodal action of the investigational anti-*Candida* lipopeptide (AF_4_) lead from *Bacillus subtilis*

**DOI:** 10.3389/fmolb.2023.1248444

**Published:** 2023-12-06

**Authors:** Swetha Ramesh, Utpal Roy, Subhashis Roy

**Affiliations:** ^1^ Department of Biological Sciences, Birla Institute of Technology and Science, K.K. Birla Goa Campus, Goa, India; ^2^ Department of Chemistry, Birla Institute of Technology and Science, K.K. Birla Goa Campus, Goa, India

**Keywords:** *Candida albicans*, lipopeptide, membrane disruption, oxidative damage, apoptosis, drug-resistance silica gel-based chromatography

## Abstract

**Background:** *Candida* species are the main etiological agents for candidiasis, and *Candida albicans* are the most common infectious species. *Candida* species’ growing resistance to conventional therapies necessitates more research into novel antifungal agents. Antifungal peptides isolated from microorganisms have potential applications as novel therapeutics. AF_4_ a *Bacillus*-derived lipopeptide demonstrating broad-spectrum antifungal activity has been investigated for its ability to cause cell death in *Candida* species via membrane damage and oxidative stress.

**Methods:** Using biophysical techniques, the secondary structure of the AF_4_ lipopeptide was identified. Scanning electron microscopy and confocal microscopy with fluorescent dyes were performed to visualise the effect of the lipopeptide. The membrane disruption and permeabilization were assessed using the 1,6-diphenyl hexatriene (DPH) fluorescence assay and flow cytometric (FC) assessment of propidium iodide (PI) uptake, respectively. The reactive oxygen species levels were estimated using the FC assessment. The induction of apoptosis and DNA damage were studied using Annexin V-FITC/PI and DAPI.

**Results:** *Bacillus*-derived antifungal variant AF_4_ was found to have structural features typical of lipopeptides. Microscopy imaging revealed that AF_4_ damages the surface of treated cells and results in membrane permeabilization, facilitating the uptake of the fluorescent dyes. A loss of membrane integrity was observed in cells treated with AF_4_ due to a decrease in DPH fluorescence and a dose-dependent increase in PI uptake. Cell damage was also determined from the log reduction of viable cells treated with AF_4_. AF_4_ treatment also caused elevated ROS levels, induced phosphatidylserine externalisation, late-stage apoptosis, and alterations to nuclear morphology revealed by DAPI fluorescence.

**Conclusion:** Collectively, the mode of action studies revealed that AF_4_ acts primarily on the cell membrane of *C. albicans* and has the potential to act as an antifungal drug candidate.

## 1 Introduction

*Candida albicans* is a predominant fungus and the most common causal agent of superficial and mucosal inflammations in systemic fungal infections (Pappas et al., 2018). This opportunistic yeast is a resident organism in the gastrointestinal, genitourinary, and upper respiratory tracts of humans and can cause both local and systemic infections when it overgrows, especially in immunocompromised patients (Gullo, 2009). While most *Candida* infections can be treated effectively with antifungal medications, some cases can cause life-threatening complications. In order to keep the pipeline of the arsenal updated for combating *Candida* infections, new leads are required. Lipopeptides are the kind of antimicrobial peptides produced by the Gram-positive bacterium *Bacillus* (Sumi et al., 2015) that have demonstrated antagonistic potential against a variety of infections and pathogenic organisms (Mannanov and Sattarova, 2001; Ongena and Jacques, 2008). One such application of this group of compounds is their ability to inhibit the growth of the fungal species *Candida*. This is largely due to their unique lipophilic structure, which allows them to form lipophilic interactions with the cell membrane of *Candida* spp. (Tabbene et al., 2011; Olfa et al., 2015; Ramachandran et al., 2018). In addition, their hydrophobic nature allows them to penetrate the cell wall and disrupt the integrity of its membrane. This makes them a powerful weapon against *Candida* infections, as they can target and damage the cell membrane (Sikorska et al., 2014), thus causing the death of the organism. Bacillomycin lipopeptides are one of the families of bioactive lipopeptides produced by the genus *Bacillus* (Tabbene et al., 2011; Zhang et al., 2013). In our previous work, we have identified antifungal lipopeptide variants similar to bacillomycins isolated from *Bacillus* *subtilis* RLID 12.1. The lipopeptides demonstrated significant activity against multiple strains of *Candida albicans* and non-albicans species. Previously, we demonstrated the inhibitory effect of AF_4_ on *Candida* non-*albicans* species, *Candida tropicalis* and *Candida* auris (Ramesh et al., 2023). In this study, we have attempted to study the physicochemical nature of the investigational compound AF_4_, its effect on yeast cells, and its mode of antifungal action on *C. albicans* SC5314 (ATCC MYA-2876). Our results suggest that AF_4_ could cause cell death mainly by membrane disruption and by causing damage via increased ROS production. Therefore, lipopeptides from *Bacillus* may be deemed promising sources of antifungal agents and hold the potential as antifungal drug candidates to be developed into a new range of antifungals that have fewer propensities towards the development of drug resistance.

## 2 Methods

### 2.1 Preparation of antifungal compounds

The antifungal lipopeptide AF_4_ was isolated and fractionated using a process that was previously described in Ramchandran et al., 2019. *B. subtilis* RLID 12.1 cultured in shake flasks for 60 h at 30°C in a media comprised of dextrose, malt extract, peptone, sodium chloride and MnSO_4_. Using HCl (6N) of appropriate volume, the crude lipopeptide was precipitated from the cell-free supernatant after 60 h. In a 1:1 ratio, *n*-butanol and sodium phosphate buffer (pH 8.0, 50 mM) were used to dissolve the crude precipitate and solvent extraction was performed with overnight mixing followed by centrifugation. The crude extract in butanol, was then evaporated completely and dry-loaded onto an adsorption column packed with silica gel (230–400 mesh, particle size 37–63 µm). A gradient of chloroform and methanol was used to fractionate the crude extract. The eluted fractions were tested for activity against C. albicans SC5314 by spot-on lawn method. Fractions exhibiting clear zones of inhibition were taken up for purification by reverse-phase (RP) HPLC using a Luna 5 μm -C18 column (250 mm × 10 mm, particle size 5 μm) at a semi-preparative scale. The fractions that showed anti-*Candida* activity were dissolved in 200 µL methanol and injected into the HPLC. Lipopeptides were purified using a gradient solvent system comprising acetonitrile and water with 0.1% trifluoroacetic acid. The gradient of acetonitrile used for purification were as follows: 0%–45% for 0–10 min at the flow rate of 1 mL/min, 45%–54% from 10 to 20 min at 0.5 mL/min, 54%–60% from 20 to 48 min at 0.5 mL/min, 60%–100% from 48 to 65 min at 1 mL/min and 100%–5% from 65 to 75 min at 1 mL/min and monitored at 210 nm. To ensure that pure fractions with single peaks were obtained, AF_4_ fractions were collected and re-chromatographed using a Luna 5 μm C18 250 mm × 4.6 mm, particle size 5 μm). Purified fractions were pooled, lyophilised and resuspended in autoclaved 10 mM sodium phosphate buffer. The concentration of the lipopeptide was estimated by the Bicinchonnic acid (BCA) method (Ramachandran et al., 2018) (Pierce ™ BCA-Protein assay kit ThermoFisher, United States). The minimum inhibitory concentrations (MICs) and minimum fungicidal concentrations (MFCs) of AF_4_ and amphotericin B (AMB) were determined for *C. albicans* SC5314 and clinical isolates of *C. albicans* (obtained from NCCPF, Mycology Division, PGIMER, Chandigarh India) according to CLSI guidelines (Clinical and Laboratory Standards Institute CLSI, 2017).

### 2.2 Analysis of structural characteristics of AF_4_

#### 2.2.1 Fourier-transform infrared-attenuated total reflectance spectroscopy

Using the Fourier-Transform Infrared Spectrometer, a spectroscopic analysis of the HPLC-purified fractions of AF_4_ was carried out to determine the distinctive characteristics of the lipopeptide (Nasir and Besson, 2012). Spots of 100 µg of the pure antifungal lipopeptide AF_4_ were dried after being dissolved in sodium phosphate buffer (10 mM, pH 7.0). The sample spectrum was obtained by averaging numerous scans produced in transmittance mode with a resolution of 4 cm^−1^ and in the range of 400–4,000 cm^−1^.

#### 2.2.2 Circular dichroism spectroscopy

The secondary structures of the antifungal lipopeptide AF_4_ were identified using far-UV CD spectroscopy. Different solvents—10 mM sodium phosphate buffer, trifluoroethanol, water, and methanol—were used to solubilize the lipopeptides. The distinctive secondary structural features of the lipopeptide in respective solvents were identified using spectra obtained in the 200–260 nm region at a 1 nm data interval using the JASCO-CD Polarimeter J-815. The scans were plotted on GraphPad Prism9 and analysed using K2D3 (Louis-Jeune et al., 2012; Janek et al., 2018).

#### 2.2.3 Nuclear magnetic resonance (NMR) analysis

A cryo probe-equipped Bruker Avance NEO 850 MHz NMR spectrometer (TIFR, Mumbai, India) was used to record the NMR spectra of the AF_4_ lipopeptide at 25°C. DMSO-d6 (NMR-grade) was used to dissolve AF_4_ (2–3 mg) in 500 μ L of 99.8% purity. Eight scans per t1 increment, 512 t1 increments, and a 1.5 relaxation delay were used to record the 2D 1H-1H TOCSY.

### 2.3 Scanning electron microscopy (SEM)

Cells suspensions with an inoculum size of ∼10^6^ CFU/mL were prepared and exposed to the antifungal compound AF_4_ at a concentration of 8 mg/L (2X MIC) overnight. As controls, *Candida* cells were cultivated without any antifungal treatment and treated with conventional antifungal amphotericin B (AMB) at 1 mg/L (2X MIC) for 3 h. The samples were prepared using glutaraldehyde as a fixative, followed by osmium tetroxide staining. Excess fixative and osmium tetroxide were washed off after each step using sodium cacodylate buffer and sterile water as described in (Semis et al., 2013; Ramesh et al., 2023). The samples were dried using gradient ethanol dehydration and subjected to critical point drying, sputter coated with gold and visualised at a magnification of 20000X using a Quanta FEG 250 (Thermo Fisher Scientific, United States) instrument.

### 2.4 Confocal laser scanning microscopy

Confocal microscopy was employed to observe the impact of pure fractions of AF_4_ on membrane integrity (Kuhn et al., 2002; Chan et al., 2011; Zhang et al., 2018). Cell suspensions were prepared as described above. Acridine orange (AO) (20 µM) and propidium iodide (PI) (5 µg/mL) were used to stain the harvested *Candida* cells for 15 min each in the dark. Excess stain was removed by phosphate buffered saline (PBS) washes and trapped between a glass slide and cover slip. The coverslips were fixed using enamel and imaged at 120X magnification (Ramesh et al., 2023). The FUN-1 dye (5 µM) was applied to a cell suspension resuspended in a glucose-supplemented HEPES solution. The cells were incubated at 30°C in the dark for 30 min (Pina-Vaz et al., 2001a). The unbound stain was washed off with the same solution, and samples were fixed on glass slides and imaged as described above. Images were acquired using the Olympus Fluoview 3000. Excitation at 490 nm and emission at 520 nm were used for AO, while PI was used with excitation and emission maxima of 535 nm and emission maximum of 615 nm respectively. A fluorescein filter set with excitation at 480 nm and emission at 530 nm was used to image cells with FUN-1.

### 2.5 Propidium iodide (PI) influx assay

The ability of AF_4_ to permeabilize membranes was determined by PI uptake in cells treated with AF_4_ (Ramani and Chaturvedi, 2000; Lee and Lee, 2015). The antifungal lipopeptide AF_4_ at concentrations of 4 and 8 mg/L and AMB at 0.5 mg/L and 1.0 mg/L were added to cell suspension (∼5 × 10^6^ CFU/mL) in RPMI-1640 and incubated for 18 h and 3 h, respectively. Cells grown in the absence of antifungals and 70% ethanol-treated cells were used as negative and positive controls for PI uptake, respectively. Cells were harvested after treatment and resuspended in PBS. The suspensions were stained with PI (5.0 µg/mL) and incubated in the dark for 20 min. The stained cells were washed with PBS and suspensions were analysed using a FACScan flow cytometer (Becton Dickinson FACS Melody) using a 488 nm laser line and 586 nm filter for PI. The forward scatter, side scatter and percentage of PI-stained cells were recorded and analysed (Hwang et al., 2011a; Hao et al., 2013; Jia et al., 2019; Li et al., 2020). Aliquots of treated and untreated samples were serially diluted, plated on Sabouraud Dextrose (SD) agar plates and incubated for 24 h at 37°C (Green et al., 1994). The colonies formed were counted to determine the reduction in CFU/mL across treatments.

### 2.6 Assessment of plasma membrane integrity

Changes to the membrane dynamics due to antifungal action were assessed by labelling fungal cell membranes with 1,6-diphenyl-1,3,5-hexatriene (DPH). A decrease in DPH fluorescence is proportional to the extent of disruption in the membrane lipid bilayer (Lee et al., 2018). Cells were treated with 4 and 8 mg/L of AF_4_ overnight and AMB (0.5 mg/L). Cells were then fixed with 0.37% formaldehyde and incubated for 30 min at 28°C. Cells were then washed and flash-frozen by dipping them in liquid nitrogen. Cells were subsequently thawed, resuspended, and labelled with 0.6 mM DPH in PBS and incubated for 45 min at 28°C. Post-incubation cells were washed with PBS and homogenized by sonication on ice (Park and Lee, 2009; Lee et al., 2018). Cells were centrifuged, and the fluorescence intensity of the supernatant was measured using a spectrofluorophotometer (JASCO FP-8500, Japan) with 350 nm excitation and 425 nm emission.

### 2.7 Reactive oxygen species (ROS) production

ROS produced upon treatment with the antifungal was determined using 2′-7′ dichloro-dihydro-fluorescein diacetate (DCFH-DA) (Sigma-Aldrich, United States) fluorescence by flow cytometry (Da Silva et al., 2014; Seyedjavadi et al., 2020). Cells suspensions prepared in RPMI-1640 were incubated overnight with AF_4_ (4, 8 and 16 mg/L) and AMB at 0.5, 1.0 and 2.0 mg/L at 37°C in shaking condition. Hydrogen peroxide (10 mM) (H_2_O_2_)-treated cells were used as positive control and untreated cells as negative control. After incubation cells were harvested by centrifugation, washed, and resuspended in PBS with 10 µM of DCFH-DA (Li et al., 2020). The cells were stained for 30 min in the dark at 37°C. The cell suspension was washed and resuspended in PBS, and DCF fluorescence was recorded using a BD FACScan Flow cytometer. The increase in the percentage of ROS-positive cells was determined by the fluorescence of treated samples in comparison with that of untreated samples.

### 2.8 Apoptosis detection

*C. albicans* cells treated with AF_4_ at 2, 4, and 8 mg/L overnight were harvested and washed in 1X PBS. The pellet was resuspended in 1 mL of 0.1 M potassium phosphate buffer (50 mM K_2_ HPO_4_, 5 mM EDTA, 50 mM DTT, 50 mM KH_2_PO_4_, and 40 mM β-mercaptoethanol) with 2.4 M sorbitol and at pH 7.2 (Hao et al., 2013; Jia et al., 2019). To the suspension, Zymolyase 20T (0.02 mg/mL) (US Biologics, United States) was added and incubated for 20 min at 35°C. The protoplasts were washed and resuspended in 100 μL permeabilization solution (0.1 M sodium citrate (pH 6.0) with 0.1% Triton X- 100), and placed on ice for 5 min. The protoplasts were washed again and stained with Annexin V- FITC/PI apoptosis detection kit (MedChem Express, United States) according to the manufacturer’s instructions (Hwang et al., 2011a; Hwang et al., 2011b; Hao et al., 2013). The cell staining data was acquired using flow cytometry and analysed using the software FlowJo V10.

### 2.9 DAPI staining

Fragmentation and condensation of nucleic acid due to the antifungal activity of lipopeptide was assessed using 4′,6-diamidino-2-phenylindole (DAPI) (HiMedia). Cells treated with lipopeptide at 2, 4 and 8 mg/L as described above were harvested and stained with 1 µg/mL DAPI for 20 min at 37°C. Cells were imaged at 200X using an Olympus FV3000 microscope (Hwang et al., 2011a; Hwang et al., 2011b; Hao et al., 2013).

## 3 Result

**FIGURE 1:**
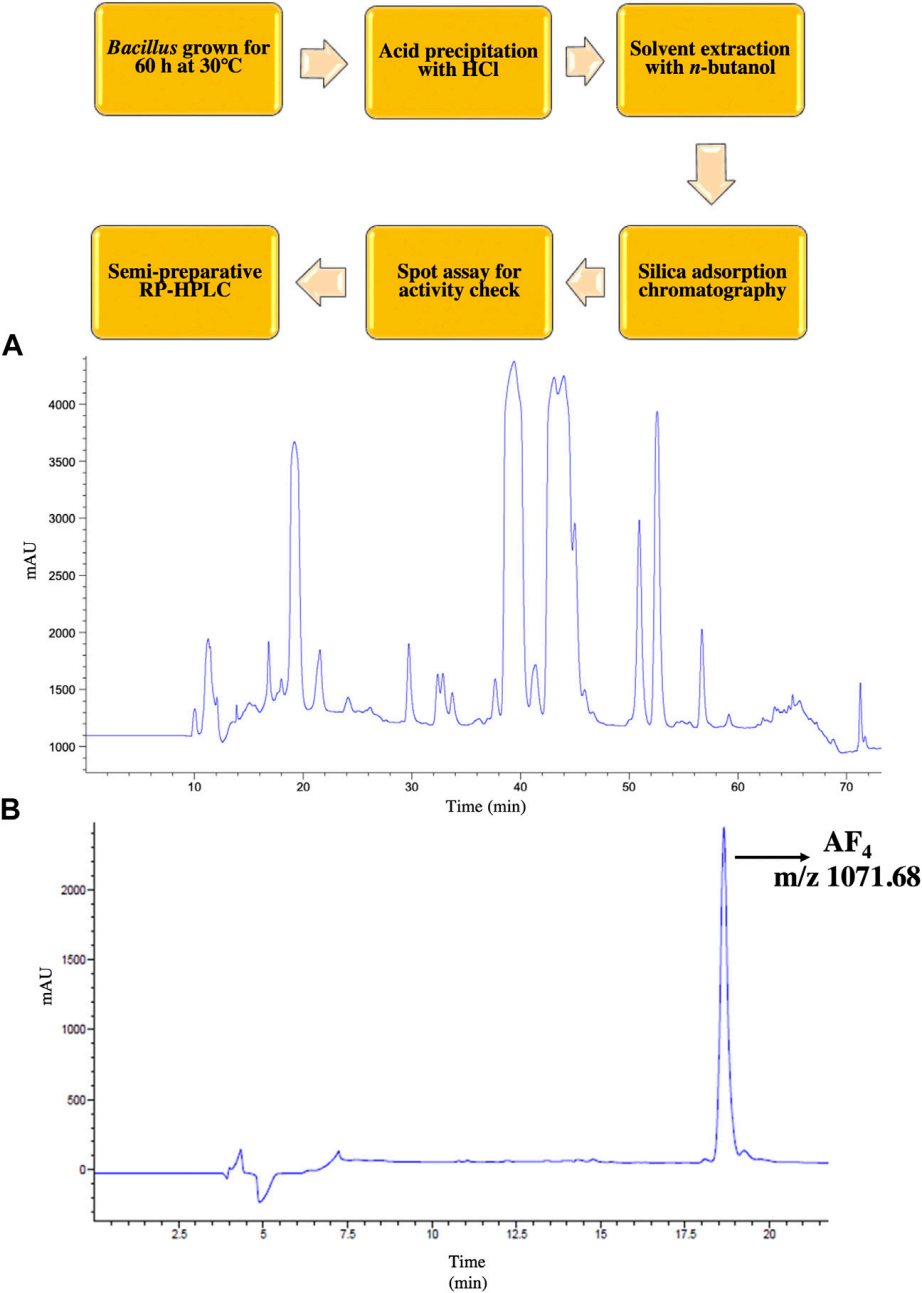
RP-HPLC purification of AF_4_. An outline of the purification scheme followed is depicted. **(A)** Chromatogram of crude lipopeptide showing 5 co-produced lipopeptide variants obtained in semi-preparative scale HPLC. **(B)** Chromatogram AF_4_ with m/z ratio 1071.68 after re-chromatography at analytical scale followed by liquid chromatography-mass spectrometry (LC-MS) showing a single peak indicative of purity of the compound.

In the present investigation, the lead lipopeptide AF_4_ was chromatographically purified to homogeneity (Figures 1A, B) from a crude mixture, as these chromatographic steps-semi-preparative (Figure 1A) and analytical scale (Figure 1B) RP- HPLC- allowed better resolution of biologically active lipopeptides with efficient fractionation (Ramachandran et al., 2018; Ramchandran et al., 2019). *C. albicans* SC5314 was inhibited at a concentration of 4 mg/L when treated with AF_4_ and at 0.5 mg/L when treated with AMB. The MIC of AF_4_ was also tested against an additional 5 clinical isolates and was found to be 4 mg/L for all strains tested (Table 1). In our previous works, MICs of AF_4_ have been tested against multiple strains of *C. albicans* and non-*albicans* and were found to be between 2 mg/L to 8 mg/L. The biocompatibility of AF_4_ was tested against NIH-3T3, RAW264.7 and Vero cell lines and showed IC_50_ values of 11.69 mg/L, 11.24 mg/L and 17.67 mg/L, respectively. The IC_50_ values were found to be more than two times the MIC values (Ramachandran et al., 2018; Ramesh et al., 2023). These studies demonstrated that AF_4_ showed low minimum inhibitory concentrations (MICs) across many fungal strains, and was found to exert low cytotoxicity at respective minimum inhibitory and fungicidal concentrations, thus indicating greater antifungal potency. Therefore, the lipopeptide, AF_4_, was chosen for further characterization of its structural features and mode of action against *C. albicans* SC5314.

**TABLE 1:** Antifungal susceptibility of *C*. *albicans* isolates tested using the AF_4_ lipopeptide and standard antifungal AMB.

Organism	AF_4_		AMB	
	MIC (mg/L)	MFC (mg/L)	MIC (mg/L)	MFC (mg/L)
*C. albicans* SC5314	4.0	4.0–8.0	0.5	1.0
*C. albicans* NCCPF 400099	4.0	4.0	0.5	1.0
*C. albicans* NCCPF 400100	4.0	4.0	0.25	0.5
*C. albicans* NCCPF 400101	4.0	4.0	0.25	1.0
*C. albicans* NCCPF 400102	4.0	4.0	0.5	1.0
*C. albicans* NCCPF 400103	4.0	8.0	0.25	1.0
*C. albicans* ATCC 24433	4.0	4.0	0.5	0.5
^*^ *C. krusei* ATCC 6258	4.0	8.0	1.0	1.0–2.0

* #- used as reference strain

### 3.1 Analysis of the secondary structure of AF_4_

#### 3.1.1 FTIR spectroscopy

**FIGURE 2:**
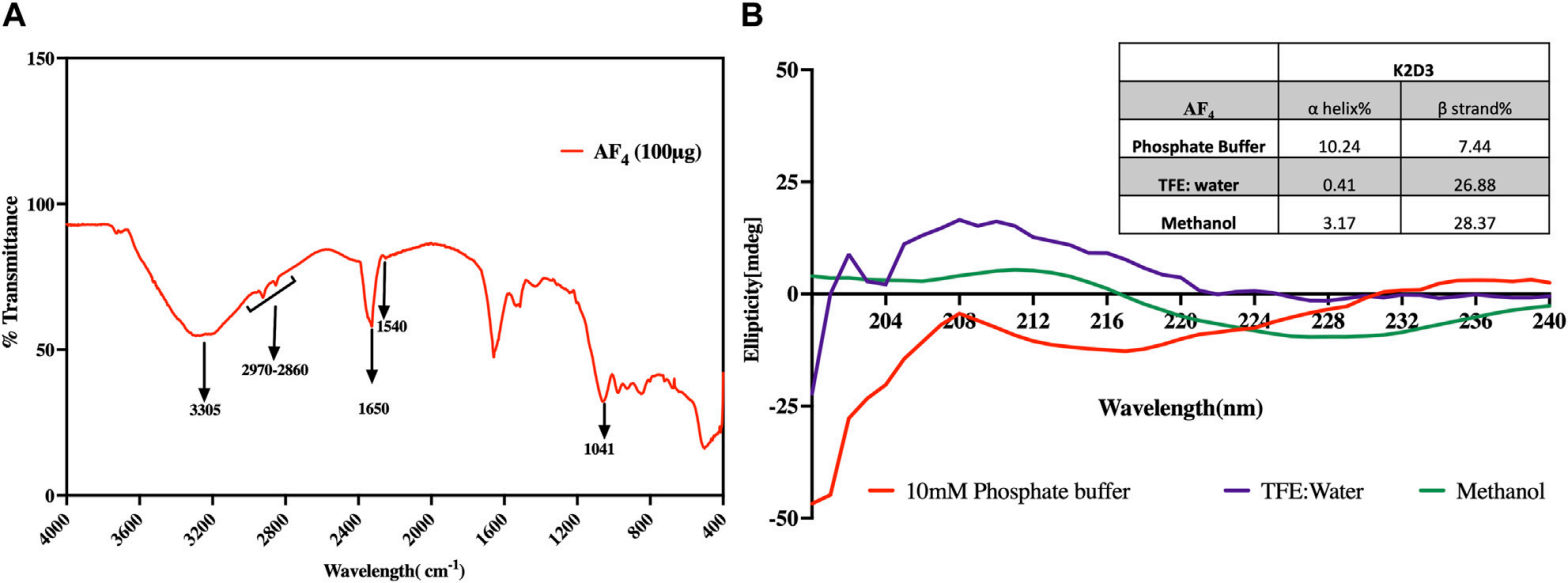
Analysis of the structural features of AF_4_. **(A)** FT-IR spectrum of purified fraction AF_4_ in the range 400–4,000 cm^−1^. **(B)** Far-UV CD spectra of AF_4_ dissolved in 10 mM Phosphate buffer, TFE diluted with water, and methanol. The inset table details the percentage of α-helix and β-strands of AF_4_ in each solvent assessed using K2D3.

The Infrared (IR) spectra of the purified fractions of AF_4_ revealed multiple bands indicative of peptides with attached lipid moieties (Figure 2A). These bands related to the N-H stretching mode and the stretching mode of CO-N belonging to the peptide components were observed at 3,305 cm^−1^ and 1,650 cm^−1^ respectively. A band at 1,540 cm^−1^ corresponding to the stretching and deformation modes of N-H bonds was also observed (Figure 2A). Additionally, bands at 1,041 cm^−1^ and 1,670 cm^−1^, corresponding to tyrosine and glutamine residues, were also detected, confirming the peptide nature of the purified fractions. Moreover, a pronounced band at 1,650 cm^−1^, representing the C=O and C=O-NH groups from the conjugation of amine groups of amino acids and fatty acid carboxylic groups, was evidence of the lipopeptide nature. Amide II components observed at 1,526 cm^−1^ were indicative of N-H hydrogen bonding and the beta-conformation of the lipopeptide. Besides, aliphatic chains related to C-H bonds associated with the lipid moiety were observed in the range of 2,970 to 2,850 cm^−1^.

#### 3.1.2 Circular dichroism

The spectra generated were analysed using K2D3 software. Predictive data suggested that the lipopeptide AF_4_ has a significant tendency to form β strands rather than α helixes (Figure 2B). The results also indicated that the lipopeptide has a significant probability of forming β strands with strong positive bands between 200 nm and 205 nm when dissolved in TFE suggesting the presence of β turns which is a characteristic of lipopeptides. Specifically, AF_4_ had a β strand probability ranging from 26% to 36% depending on the solvent used (inset Table in Figure 2B).

#### 3.1.3 NMR spectra analysis

The ^1^H NMR results obtained for the purified lipopeptides at 800 MHz indicated that the purified antifungal compound AF_4_ is a lipopeptide due to the presence of a peptide backbone (N–H at 8.0–7.2 ppm), an aliphatic carbon–hydrogen bond (C–H at 5.0–4.0 ppm), and a long aliphatic chain (CH_2_ at 1.55–1.25 ppm).

For AF_4_ lipopeptide, seven α-protons (δ_H_ 4.525, 4.518, 4.446, 4.436, 4.428, 4.177 and 4.168) of peptide bonds, a long fatty-acid chain (δ 2.298–1.127), terminal methyl groups (δ_H_ 0.861–0.852), and a peptide backbone (eight amide protons, δ_H_ 8.728–7.001) were observed in the compound AF_**4**_ in the ^**1**^H-NMR spectrum (Supplementary Figure S1)**.** Chemical shifts at 0.85 (^**1**^H-NMR)/14.43 in the ^13^C-NMR (Supplementary Figure S2) indicate the presence of the CH_3_ group indicating the probable triplet methyl signal at δ_H_ 0.852–0.861. A β-amino acid where the *β*CH (β protons) at *δ*4.03 ppm might be connected with an NH at 7.156 or 7.137 ppm and with two pairs of protons belonging to CH groups at 2.349 and 1.412 ppm (in ^13^C-NMR) in AF_4_ as indicated by TOCSY (Supplementary Figure S4) spectra. Among the seven amino acid residues, one is a tyrosine (with reference to a pair of doublets around 7.15 ppm) and a side-chain amide group (either Asn or Gln), where each of these residues formed a pair of coupled signals between 8.07 and 8.72 ppm. The backbone amide of L-asparagine (Asn) at δ_H_ 4.44 ppm correlates to the β-amino acid α-protons at δ_H_ 2.28 ppm and to the β-proton at δ_H_ 4.03 ppm. ^13^C carbon signals (Supplementary Figure S2) at *δ* 56.66 (α-CH), 115.54, 128.42, 130.23 and 156.28 were assigned to the aromatic carbons of the Tyr residue.

The two-dimensional AF_4_ COSY (Supplementary Figure S3) ^1^H NMR demonstrated the presence of a β-amino fatty acid, with the resonance of the βC proton appearing at δ 4.46 ppm. This proton possibly got coupled to the α-C protons of the fatty acid which resonated at δ 2.0 ppm, and also to the adjacent protons of the fatty acid chain at δ1.7 ppm. The ^1^H NMR spectrum of AF_4_ revealed the presence of a long aliphatic chain (CH2)_n_ which was observed between δ_H_ 1.24–1.41 ppm and CH_3_ at 0.85/0.86 ppm. Seventeen carbon resonances were resolved for the β amino acid. The ^13^C NMR spectrum of the AF_4_ lipopeptide revealed seven carbonyl carbons in the characteristic range *δ*C 170.75–173.81. The overlapped carbon signals exhibited in the upfield δ_H_ 0.85–0.86 in the ^1^H NMR spectrum and a cluster of methylene carbon signals around δ_H_29.0 (29.08–29.58) in the ^13^C NMR indicated a long chain alkyl group existing in the AF_4_ lipopeptide.

The carbon signals at ppm 40.1/40.2 (methylene) and βCH_2_ 45.827 (methine) are typical to β-amino fatty acid with a long side chain and could be assigned to the α -CH2 (C-34) and *j*8-CH (C-33, adjacent to NH) of the long-chain β-amino acid. AF_**4**_ TOCSY spectrum (Supplementary Figure S4) identified six spin systems corresponding to 6 amino acids. For Gln and Asn, a TOCSY peak between 2 amine protons between the chemical shift of 6.9 and 7.6 ppm was observed; besides, the TOCSY spectra revealed amino acid peaks of Tyr, Asn, Gln, and Ser residues. However, two peaks in TOCSY spectrum could not be assigned to any amino acid residue due to ambiguities or lack of clarity. The TOCSY spectra for AF_**4**_ revealed 6 spin systems, and 21 protons, and indicate the presence of β amino acid with an aliphatic character with five correlations to an amide proton to resonate at *δ* 7.157 ppm.

### 3.2 Field-emission scanning electron microscopy (FE-SEM)

**FIGURE 3:**
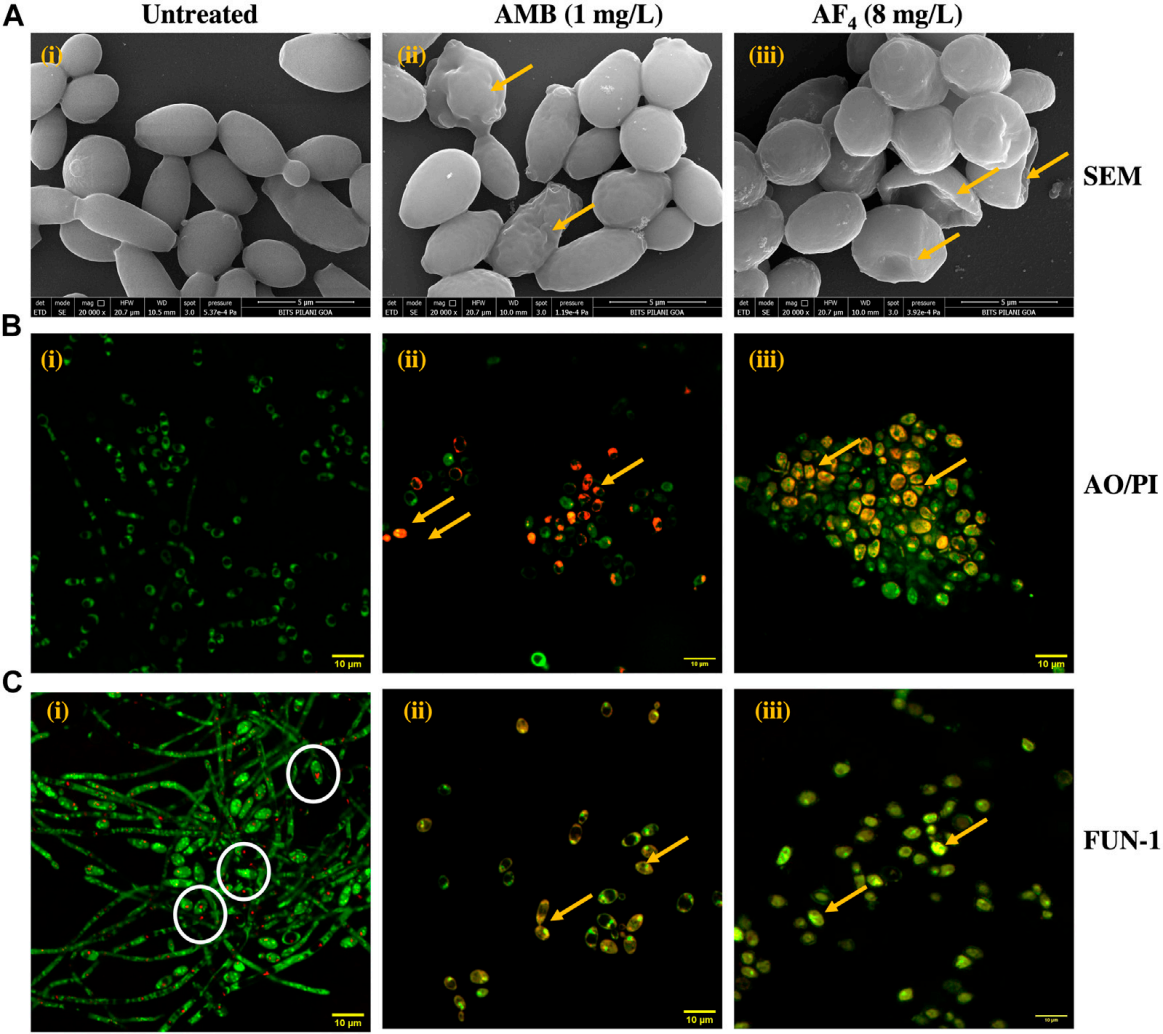
Visualization of antifungal effect of AF_4_. **(A)** Scanning electron micrographs of *C. albicans* SC5314 cells: (i) Untreated, (ii) Treated with 1 mg/L AMB and (iii) Treated with 8 mg/L of AF_4_. Magnification of 20000X. Scale bar 5 μm. Yellow arrows indicate regions of membrane damage visible on the cell surface. **(B)** Confocal microscopy images of AO/PI stained *C. albicans* SC5314 cells with (i) Untreated, (ii) AMB 1 mg/L, and (iii) AF_4_ 8 mg/L. Magnification of 120X. **(C)** Confocal images of *C. albicans* SC5314 cells stained with FUN-1: (i) Untreated, (ii) Treated with 1 mg/L AMB and (iii) 8mg/L AF_4_. Magnification of 120X. White circles show CIVS with red fluorescence formed in the untreated cells. Yellow arrows indicate regions of membrane damage where dye uptake can be visualized.

SEM was performed to elucidate the ultrastructural alterations of *C. albicans* SC5314 cells treated with the antifungal lipopeptide AF_4_ and the standard polyene AMB. Results of SEM after overnight treatment with antifungal AF_4_ and 3 h treatment with AMB (Figure 3A) revealed that cells treated with the lipopeptide AF_4_ showed damages on the cell surface, comparable to that caused by antifungal AMB, observed as indents, dimples, and concavities indicated in Figure 3A by yellow arrows, whereas cells grown in the absence of antifungals maintained their ovoid cell shape with smooth surface and bud scars.

### 3.3 Confocal microscopy of *Candida* cells

Confocal microscopy was used to examine the influence of the antifungals AF_4_ and AMB on the permeability and metabolic state of *C. albicans* SC5314 cells. When treated with AO/PI, as seen in Figure 3B, only green fluorescence was observed in the control panels, indicating intact membranes permeable only to AO (Zhang et al., 2018; Fiołka et al., 2021). However, cells exposed to AMB (used as a positive control) and AF_4_ showed reddish-orange fluorescence due to PI uptake, indicating cell membrane damage and permeabilization.

FUN-1 is a two-colour fluorescent probe that utilizes the metabolic pathways that are common among yeasts. The biochemical conversion of FUN-1 dye’s diffuse green fluorescence into condensed orange-red aggregates within the vacuoles (CIVS) in healthy cells is proof of intact cell membranes and active metabolism (Millard et al., 1997). In contrast, cells affected by the antifungal molecule show green-yellow fluorescence distributed throughout the cell. As depicted in Figure 3C, untreated cells remained viable and demonstrated the formation of red fluorescent CIVS. On the contrary, cells treated with AMB and AF_4_ showed yellow-green fluorescence, signifying a loss of metabolic activity and increased membrane permeability.

### 3.4 Flow cytometric analysis of PI influx

**FIGURE 4:**
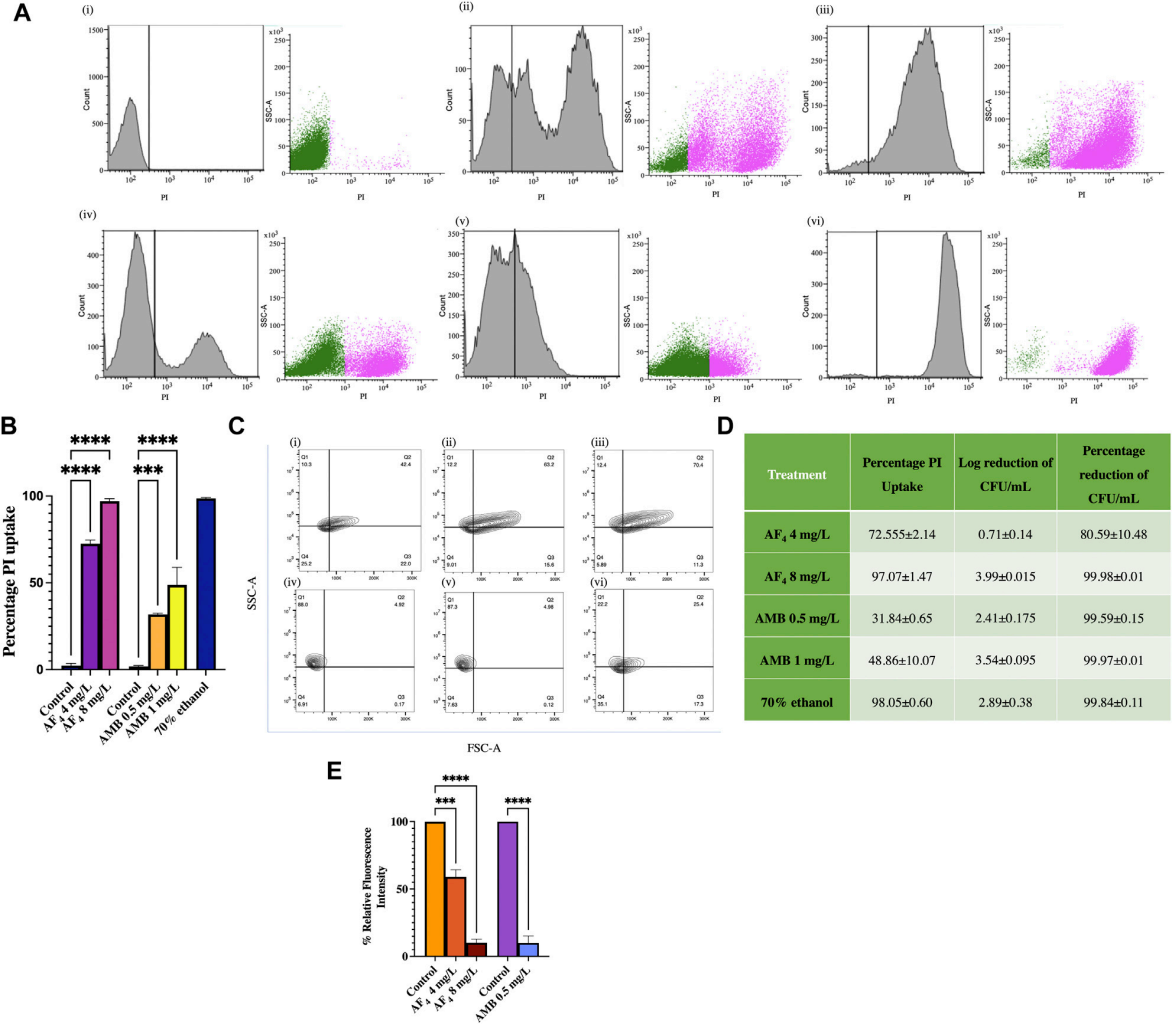
Assessment of membrane integrity. **(A)** Histograms of PI uptake observed in *C. albicans* SC5314 (i) Untreated, (ii) AF_4_ 4 mg/L, (iii) AF_4_ 8 mg/L, (iv) AMB 0.5 mg/L, (v) AMB 1 mg/L and (vi) 70% ethanol. Cells stained with PI were analysed using a 488 nm laser with a PI filter (586 nm) and 30,000 events were recorded. **(B)** Percentage PI uptake of cells treated with AF_4_, AMB and 70% ethanol. **(C)** *C*. *albicans* SC5314 treated with AF_4_ and AMB: FSC; forward scatter and granularity SSC; side scatter after treatment. The percentage depicted is indicative of cells falling in each quadrant Q1: FSC-A−, SSC-A+ Q2: FSC-A+, SSC-A+ Q3: FSC-A+ SSC-A− Q4: FSC-A- SSC-A−. Panels are (i) Untreated cells, (ii) AF_4_ (4 mg/L), (iii) AF_4_ (8 mg/L), (iv) AMB (0.5 mg/L) (v) AMB (1 mg/L) and (vi) 70% ethanol-treated. **(D)** PI uptake percentages and corresponding log reduction values of CFU/mL. The percentage of PI uptake shown is averaged across two experiments. **(E)** Relative fluorescence intensity of DPH-labelled cells after treatment with AF_4_ (4 and 8 mg/L) and AMB (0.5 mg/L). All experiments were performed in duplicate, and the data represented as mean ± SD of results with One-way ANOVA *p*-value < 0.0001.

Flow cytometry was utilised to measure the altered permeability of the cell membrane using the vital stain PI (Pina-Vaz et al., 2001b), following treatment with lipopeptide AF_4_. Treatments with AF_4_ and 70% ethanol resulted in a greater fluorescence of PI uptake than untreated cells, which showed only negligible PI fluorescence (Figure 4A). Scatter plots in Figure 4A describe the side scatter (pink dots) increase corresponding to antifungal treatment, indicating an increase in the granularity of the cells analysed. Colony counts of the lipopeptide treatment aliquots plated correlated well with the PI uptake percentages. For AF_4_, the percentages of PI uptake were recorded as 72.5% ± 2.14% and 97% ± 1.47% when treated with 4 and 8 mg/L of AF_4_, respectively. For AMB, the uptake percentage was observed to be 31.84% ± 0.65% and 48.86% ± 10.07% at 0.5 mg/L and 1 mg/L after 3 h of treatment. In comparison, cells treated with ethanol showed near-complete membrane damage, with 99% PI uptake. The number of colonies from the aliquots of the lipopeptide treatments correlated well with the percentages of PI uptake (Figure 4B).

Changes to cell surface granularity were observed from the FSC-A vs. SSC-A contour plots, an increase in SSC-A indicated increased membrane granularity (Figure 4C). Increased distribution in quadrant one (Q1-FSC-ve/SSC + ve) was seen in cells treated with AF_4_ (Figures 4C(ii), (iii)) in comparison to untreated cells (Figure 4C(i)) indicating an increase in the granularity. For the cells treated with AMB (Figures 4C(iv), (v)) a reduction in distribution was observed in Q3(FSC +ve/SSC-ve) indicating reduced cell size and shrinkage and an increase in Q1 (Q1-FSC-ve/SSC+) was observed indicating increased granularity.

The reduction in cell numbers after treatments with AF_4_ and AMB was estimated from the colony counts obtained by plating serial dilutions of aliquots of the samples treated with 4 and 8 mg/L of AF_4_ and 0.5 and 1 mg/L of AMB on SD agar plates. The number of colonies was recorded after a 24-h incubation, and percentage reduction and log reduction values were determined. The percentage reduction in colony count of *Candida* cells treated with 4 and 8 mg/L of the AF_4_ lipopeptide was found to be 80.59% (log reduction of 0.71 ± 0.14) and 99.98% (log reduction of 3.99 ± 0.015), respectively (Figure 4D). Treatment with AMB resulted in a 99% reduction in CFU numbers at both concentrations tested showing CFU log reduction of 2.41 ± 0.175 and 3.54 ± 0.095 for 0.5 and 1 mg/L respectively.

### 3.5 Plasma membrane integrity measured using DPH fluorescence

When the membrane is bound with DPH, the reduction in fluorescence intensity indicates the membrane perturbation of the *C. albicans* plasma membrane, mediated by the lipopeptide AF_4_. The antifungal effect results in the disruption of lipid bilayer organisation and restricts the binding and subsequent fluorescence intensity of DPH. Treatment with AF_4_ at 4 mg/L and 8 mg/L led to a reduction in relative fluorescence intensity to 59.06% ± 5.31% and 10.16% ± 2.76%, respectively in comparison to the fluorescence intensity observed in untreated cells (Figure 4E). Treatment with AMB at 0.5 mg/L showed a relative fluorescence intensity of 9.93% ± 5.22%.

### 3.6 Evaluation of ROS production

**FIGURE 5:**
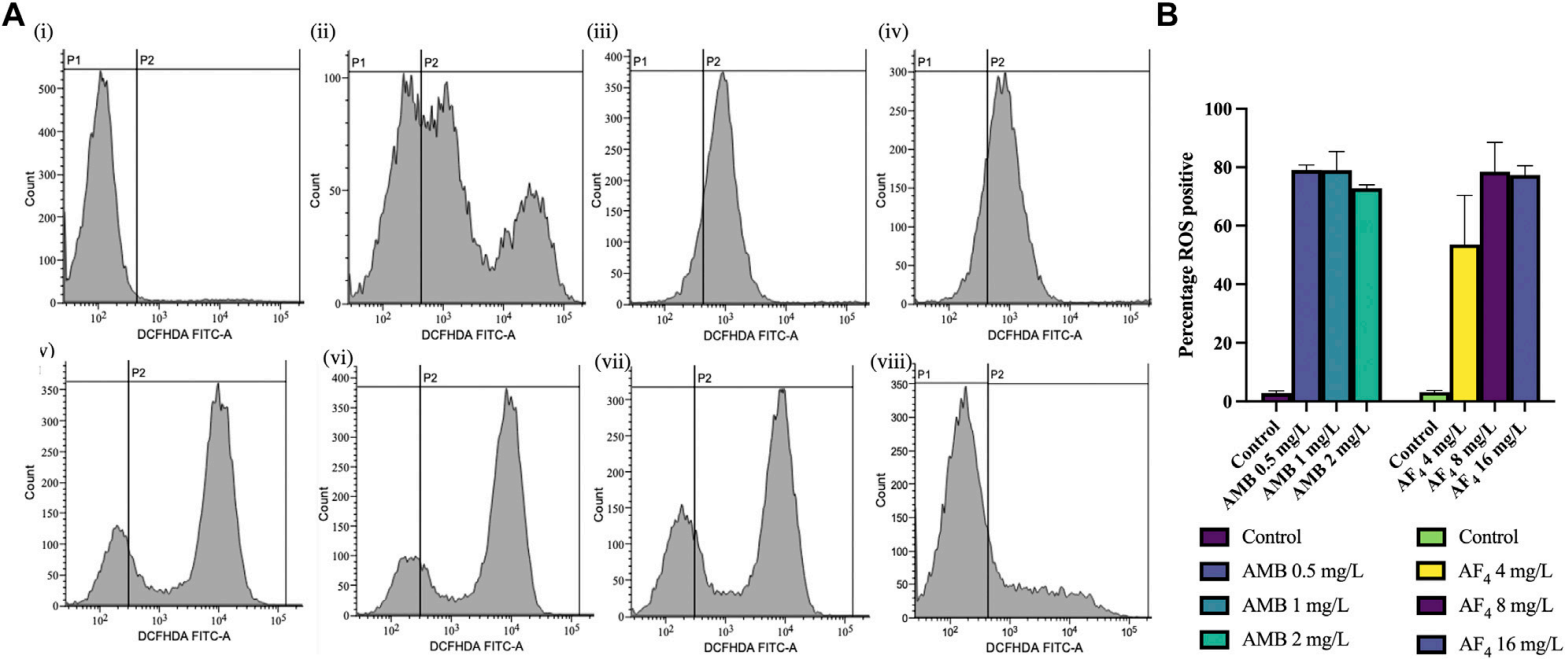
ROS analysis. **(A)** FC histograms of *C. albicans* SC5314 cells stained with DCFH-DA, analysed using a FITC filter. Panels showing DCFH-DA positive cells (FL1) indicating the ROS generated by following treatments (i) Untreated cells, (ii) AF_4_ (4 mg/L), (iii) AF_4_ (8 mg/L) (iv) AF_4_ (16 mg/L) and (v) AMB—0.5 mg/L, (vi) AMB 1 mg/L, (vii) AMB 2 mg/L and (viii) 10 mM H_2_O_2_. **(B)** Percentage of ROS-positive cells in *C. albicans* across treatments. Results represent the mean ± SD of experiments performed in duplicate, with One-way ANOVA *p*-value < 0.0001.

The generation of ROS is an important marker of antifungal activity and of cells undergoing early apoptosis (Hao et al., 2013; Da Silva et al., 2014; Jia et al., 2018). To assess the influence of antifungal lipopeptide AF_4_ on endogenous ROS levels, the intensity of fluorescence caused by the oxidation of the cell-permeable fluorescent probe DCFH-DA to DCF was estimated. Results showed that AF_4_-treated *C. albicans* cells displayed significantly elevated levels of ROS (Figure 5A) in comparison to untreated cells, with values of 53.57, 78.44, and 77.30% (Figure 5B) for concentrations of 4, 8, and 16 mg/L, respectively. Additionally, AMB-treated cells showed a significant increase in fluorescence (Figure 5A), with more than 70% ROS-positive cells observed at all concentrations of AMB used (Figure 5B). ROS is considered a key factor in the induction of apoptosis in yeast, and hence exposure to phosphatidylserine (PS) and DNA damage were investigated.

### 3.7 PS externalisation and induction of apoptosis

**FIGURE 6:**
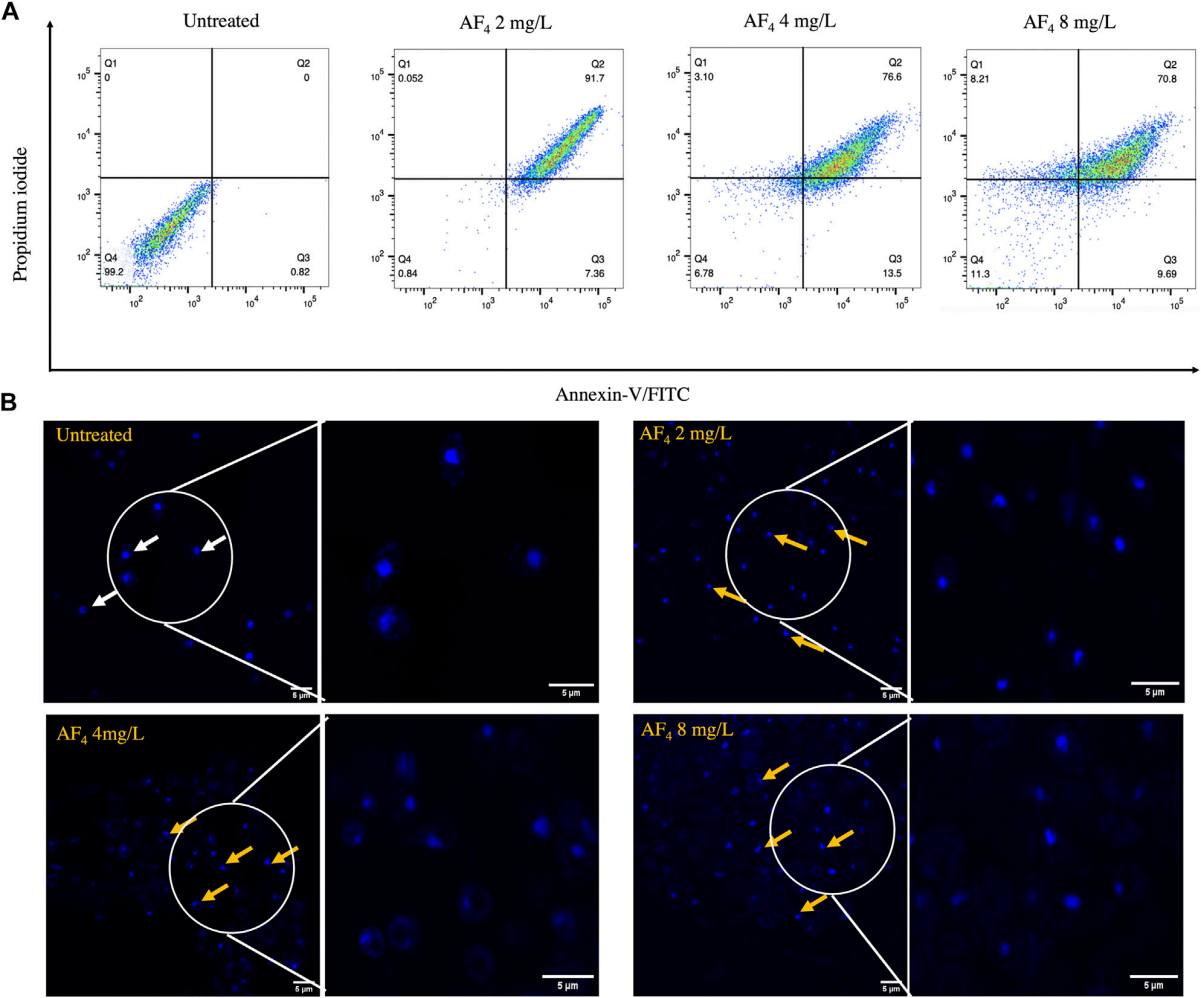
Apoptosis and DNA damage. **(A)** Annexin V-FITC/PI staining of *C. albicans* cells treated with AF_4_ lipopeptide. The cell populations are described in quadrant dot plots: Q1: necrosis (Annexin V−/PI+), Q2: late apoptosis (Annexin V+/PI+), Q3: early apoptosis (Annexin V+/PI−), and Q4: viable cells (Annexin V−/PI−). 10,000 events were collected and analysed using FlowJo V_10 (38). **(B)** Confocal images of *C. albicans* SC5314 cells stained with DAPI post-treatment with antifungal lipopeptide AF_4_. White arrows indicate intact rounded nuclei, and yellow arrows indicate nuclei that have lost normal morphological features and appear to have tube-like, teardrop shapes. DAPI preferentially stains dead cells and hence shows increased DAPI brightness compared to untreated cells (Wallberg et al., 2016). Right panel depicts magnified image of the encircled area.

Induction of apoptosis results in the translocation of PS from the inner to the outer leaflet of the plasma membrane (Da Silva et al., 2014; Crowley et al., 2016; Li et al., 2019). Apoptosis induced by lipopeptide treatment was measured by flow cytometry using Annexin V-FITC and PI double staining. Annexin V-FITC stain binds to externalised PS and is a marker for early apoptosis, and PI binds to membrane-compromised cells, indicating necrotic cells. *C. albicans* SC5314 cells after treatment with lipopeptide AF_4_ were mostly detected in quadrant 2-corresponding to Annexin V-FITC, and PI-positive (Figure 6A) (Crowley et al., 2016), which indicates late-stage apoptosis or cell death. Untreated cells were distributed in quadrant 4, negative for the uptake of both stains and hence indicating the absence of PS exposure and membrane damage. A shift towards necrosis (Figure 6A) was observed at the fungicidal concentration of 8 mg/L, consistent with previously obtained results.

### 3.8 Nuclear damage assessed by DAPI staining

DAPI, a membrane-penetrable dye that fluoresces upon binding to nucleic acids, enabled the visualisation of nuclear morphology and DNA damage, fragmentation, or condensation. Cells treated with AF_4_ at different concentrations exhibited abnormal nuclei possessing tubular, tear-drop shaped or distorted structures, as seen in Figure 6B, denoted by yellow arrows. Meanwhile, untreated cells, indicated by white arrows, demonstrated spherical nuclei with normal morphology.

## 4 Discussion

Lipopeptides are reported to destabilize membranes and modulate membrane-associated functions like permeability, leading to cell death in the *Candida non-albicans* strain/isolate (Ramesh et al., 2023). Makovitzki et al. (2006) investigated the antimicrobial activity of a series of ultra-short peptides, four amino acids long with a lipophilic tail, which had antimicrobial activity against a series of common bacterial and fungal pathogens, with varying degrees of efficacy.

AF_4_, a lipopeptide, was observed to have low MICs across several fungal strains and exhibited notable antifungal potency. To reach clinical use, the structure and mode of action of AF_4_ must be investigated. Previous studies have shown that this lipopeptide can destabilize membranes and modulate membrane-associated functions, such as permeability, which results in cell death in *Candida* non-*albicans* strains. This offers many advantages over existing antifungals in terms of their mechanisms of action and cellular targets, such as fungal membranes, cell wall and cell cycle components, and nucleic acids.

The amphipathic structure of short lipopeptides, which is composed of positively charged amino acid residues and fatty acid chains, is a key feature of antibiotic peptides. Additionally, the hydrophobic tail and hydrophilic head of these lipopeptides determine their surface-active properties. CD, FT-IR, and NMR were used to gain insight into the secondary structure of lipopeptide AF_4_. The FTIR study showed two component bands at 1,696 and 1,650 cm^−1^, which correspond to the peptide backbone (Figure 2A). AF_4_ IR spectra had features that were typically found in the IR spectra of other reported *Bacillus*-derived lipopeptides (Mukherjee and Das, 2005; Mukherjee et al., 2008; Pueyo et al., 2009; Sivapathasekaran et al., 2009). The presence of β turns evidenced by CO-N stretching peaks centred around 1,650 cm^−1^ similar to those observed in the FTIR spectra of bacillomycins D and Lc were also seen in the spectra of AF_4_ (Nasir and Besson, 2012).

The CD spectra (Figure 2B) revealed a negative band at ∼198 nm and a broad positive band centred at 208 nm in all solvents. The NMR data indicated that the peptide moiety contained seven amino acids per molecule and a long-chain fatty acid. Malina and Shai (2005) suggested that the antifungal activity of lipopeptides is dependent on the length of their aliphatic chain, with longer chains (10–16 carbon atoms) forming oligomers in the plasma membrane and creating pores. Similar to the CD spectra of iturins, the different conformations of lipopeptides such as the positive mean ellipticity values obtained around 190 nm and 210 nm were seen only in TFE but absent in buffer (Besson et al., 1996). Greater variability is observed in the CD spectra of peptides with β sheet than those with predominantly α-helix conformations and are also affected by solvents, environment and challenging the accurate determination of side chains of the peptide moiety from CD spectral data (Dang and Hirst, 2006). Iturins and iturin-like peptides such as mycosubtilin (Besson et al., 1976; Peypoux et al., 1986), bacillomycin L (Besson et al., 1977), bacillomycin D (Peypoux et al., 1980), bacillomycin F (Mhammedi et al., 1982) and mojavensin A (Ma et al., 2012) form a multigene family. The first three amino acids are shared within this family of lipopeptides whereas the remaining four are variable. Iturinic lipopeptides are often produced as a mixture of compounds with related alkyl tails forming different alkyl isomers, e.g., C14, C15, and anteiso-C15 (Hourdou et al., 1989; IWASE et al., 2009).

The MS/MS analysis showed that the isolated lipopeptide contains Asn-Pro-Tyr-Asn-Gln-Thr-Ser-long chain β-amino acids in its peptide sequence followed by a 3-amino-15-methylhexadecanoic acid with an m/z ratio of 1,071.68. The presence of methyl chain and alkyl groups was also observed from the NMR spectra. The ^1^H-NMR (Supplementary Figure S1) spectra showed the presence of seven α-protons of peptide bonds, fatty acid chain and methyl groups with δ values of approximately 0.84. The ^13^C NMR (Supplementary Figure S1) suggested the presence of 9 carbonyl groups and α-carbons or β-carbons of seven amino acids in the typical range. Two signals, 42.22 (methylene) and CH_2_ 45.82 (methine), found to represent the characteristic β-amino fatty acid residue of lipopeptides similar to iturinic lipopeptides were in agreement with previous study (Kajimura et al., 1995). A cluster of methylene carbon signals at H 29.0 (29.08–29.51/29.58) in the ^13^C NMR spectrum and carbon signals in the upfield H 0.85–0.86 in the ^1^H NMR spectrum showed a long chain alkyl group may imply branch at the end of the fatty-acid chain similar to previous study (Eshita et al., 1995). AF_4_ was found to have a long alkyl chain with 17 carbons (C17). Five amino acids of the seven amino acids Asn (2 ×), Ser (1), Gln (1) and Tyr (1) were identified by sequential walking along the TOCSY and NOESY (Supplementary Figure S4) spectra.

Cell suspensions were treated with 1X (4 mg/L) and 2X (8 mg/L) of the AF_4_ to observe the dose-dependent effect of the antifungal lipopeptide at minimum inhibitory and minimum fungicidal concentrations. The electron microscopy images showed that treatment with AF_4_ caused changes in the ultrastructure, including wrinkles and indentations (Figure 3A). These surface changes were like those observed with the antifungal AMB and could lead to a distortion of the cell membrane and a decrease in cell viability. The images indicate that the AF_4_ lipopeptide might cause damage in a way similar to iturinic peptides (Maget-Dana and Peypoux, 1994), probably by creating a gradual thinning and eventual disruption of the membrane.

Confocal laser scanning microscopy was employed to discern the effect of AF_4_ on membrane integrity. AO fluoresces green due to binding to dsDNA in cells with intact membranes, whereas PI can pass through cells with compromised membranes or nonviable cells only. We observed that live nucleated cells fluoresce green, and lipopeptide treatment allows *Candida* cells stained with AO and PI to fluoresce red due to quenching, signifying permeabilized membranes and cell death (Figure 3B). Additionally, membrane permeability and cell viability upon lipopeptide treatment were examined with the chlorinated cyanine dye FUN-1 (Figure 3C). The AF_4_-treated cells convert FUN-1 into the characteristic CIVS, suggesting that the metabolic activity of *Candida* cells is reduced post-treatment. This suggests that AF_4_ is able to permeabilize the cell membrane enough to allow the entry of the membrane-impermeable fluorescent stains and the subsequent loss of metabolic function. These events may potentially lead to cell death.

Cell membrane kinetics and loss of membrane integrity upon lipopeptide treatment were also determined using the fluorescent dyes DPH and PI (Ma et al., 2020). DPH intercalates within the hydrocarbon tails of phospholipids in intact membranes without disruption. Treatment with AF_4_ showed a dose-dependent decrease in DPH fluorescence in comparison to untreated cells, indicating a loss of membrane integrity. The disruption of the plasma membrane due to AF_4_ was further assessed by flow cytometry (Ramani et al., 1997; Ma et al., 2020). The percentage of PI uptake by cells showed increasing PI uptake when treated with an increasing dosage of AF_4_, and plate counts showed a nearly 99% reduction in CFU/mL, demonstrating a correlation between membrane damage inferred from PI uptake and cell death. The PI uptake and CFU reduction data were in good agreement, suggesting that membrane disruption plays a key role in the antifungal action of the lipopeptide.

Several studies showed that antifungal agents such as AMB, miconazole, and itraconazole exert their action (Kobayashi et al., 2002; Giudici et al., 2006; Aerts et al., 2007; Mesa-Arango et al., 2012; Delattin et al., 2014; Shekhova et al., 2017; Li et al., 2020) by producing increased ROS levels. Previous studies have shown that antimicrobial peptides such as plant defensins, cecropins, histatins, and human lactoferrin result in endogenous ROS production (Struyfs et al., 2021). Antifungal cyclic lipopeptides such as surfactins, fengycins, and tyrocidines from different *Bacillus* species (Troskie et al., 2014; Zhang and Sun, 2018; Wu et al., 2019), which appear to be similar to the AF_4_ lipopeptide, were also observed to accumulate endogenous ROS and trigger apoptotic pathways. ROS is generated as a natural by-product of cellular processes, but an increase in ROS due to stress can result in oxidative damage to cellular components. We have observed that increasing concentrations of AF_4_ result in an increased number of ROS-positive cells (Figures 5A, B). Whereas a decrease in ROS-positive cells was recorded when treated with AMB at higher concentrations (Figures 5A, B). The decrease could be attributed to cell death at higher concentrations of AMB and therefore an absence of ROS. The elevated ROS levels can contribute to increased cell membrane permeability and nucleic acid damage, eventually causing cell death. The excessive ROS produced damages nucleic acids, proteins, and lipids and may also cause membrane damage and permeability (Hwang et al., 2011a; Maurya et al., 2011; Redza-Dutordoir and Averill-Bates, 2016; Seyedjavadi et al., 2020). Several types of stresses may also cause a disturbance to the cellular homeostasis, resulting in ROS generation, making it difficult to determine the exact cycle of events leading to cell death after antifungal treatments. Further studies will be required to clarify the sequence of events involving membrane damage and oxidative stress that leads to necrosis, apoptosis and ultimately cell death.

ROS production plays a key role in the induction of apoptosis. Apoptosis is characterized by the exposure of PS on the cell surface and DNA damage, fragmentation, and condensation (Madeo et al., 1997; Phillips et al., 2003). Exposure of PS to the outer leaflet of membranes and cell death due to the antifungal action of AF_4_ were observed using an apoptosis detection kit (Hwang et al., 2011a; Hwang et al., 2011b; Hwang I. et al., 2011). The cells treated with AF_4_ were observed to be positive for Annexin V-FITC and for PI as an indication of the exposure of PS and the membrane permeabilizing effect of AF_4_. It was observed that most cells were in the late stages of apoptosis, with a shift towards necrosis proportional to AF_4_ dosage. To further investigate the presence of DNA damage, a hallmark of late apoptosis, cells treated with the antifungal were stained with DAPI (Hao et al., 2013). We observed a loss of typical nuclear morphology after drug treatment, the nuclei were observed to be tube-like, or teardrop-shaped in comparison to the rounded nuclei seen in untreated cells. The nuclear damage observed may be a direct result of AF_4_ activity or a result of ROS generated within the cells upon lipopeptide treatment.

## 5 Conclusion

In conclusion, this study presents the antifungal mode of action of *Bacillus*-derived lipopeptide AF_4_ against *C. albicans*. The collective results presented here indicate that AF_4_ has a combined mode of action that involves disruption of membrane integrity, membrane permeabilization, ROS generation, and PS externalisation, leading to late-stage apoptosis with necrosis and cell death. Additionally, since AF_4_ is minimally cytotoxic, and has a broad spectrum of antifungal activity (Tabbene et al., 2011; Ramchandran et al., 2019), it may have certain advantages for use as an antifungal lead molecule in drug development, with a low possibility of developing drug resistance against such a lipopeptide.

## Data Availability

The authors acknowledge that the data presented in this study must be deposited and made publicly available in an acceptable repository, prior to publication. Frontiers cannot accept a manuscript that does not adhere to our open data policies.
