# Supplementary material for: The elucidation of the multimodal action of the investigational anti-Candida lipopeptide (AF4) lead from Bacillus subtilis
Source: Front Mol Biosci. 2023 Dec 6;10:1248444. doi: 10.3389/fmolb.2023.1248444 (PMC10736182; doi:10.3389/fmolb.2023.1248444)
Supplement: Supplementary file 1 [file DataSheet1.pdf]

SUPPLEMENTARY FIGURES

AF$_4$ Proton NMR:

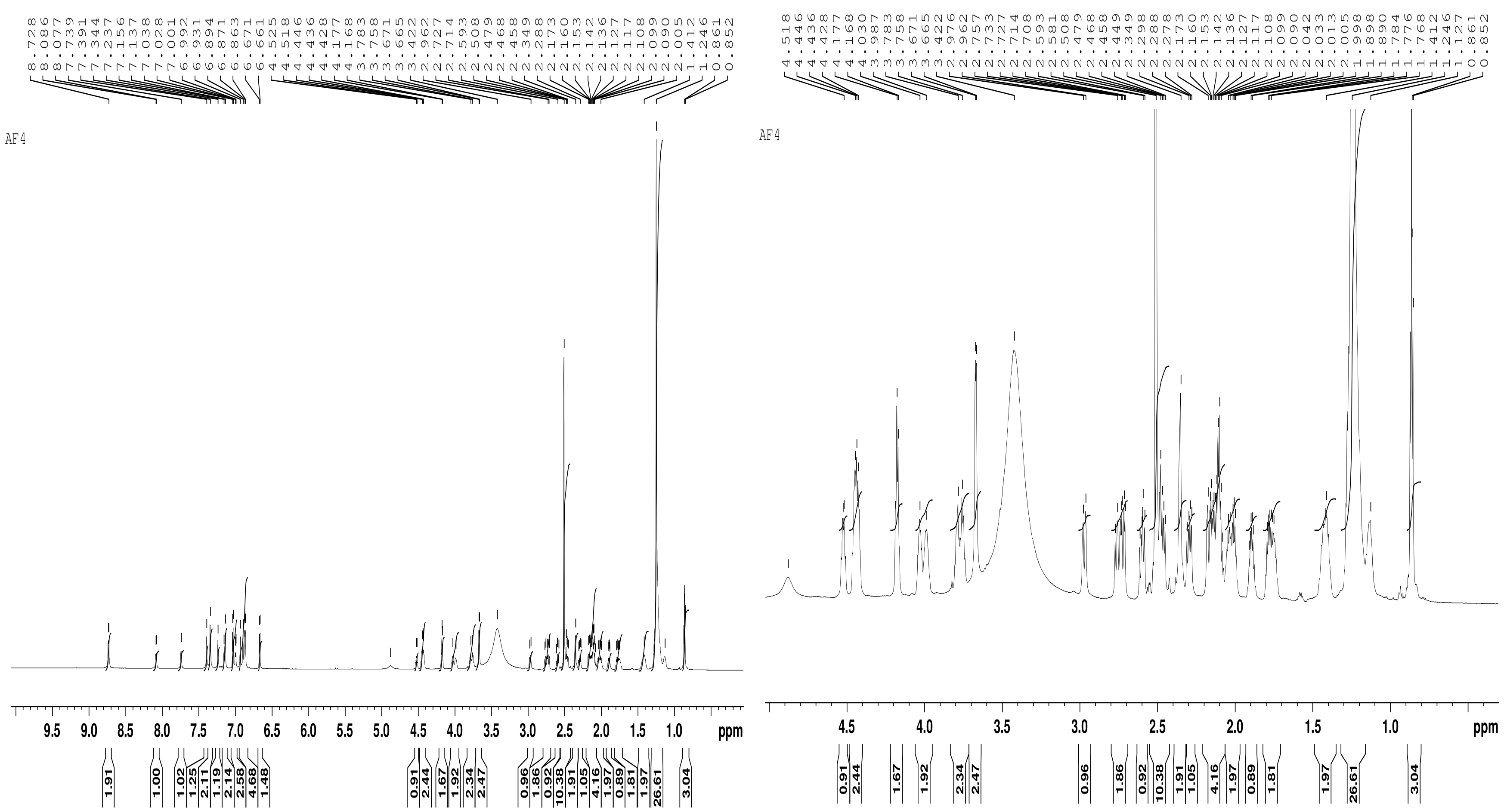

Supplementary Fig. S1.: 800 MHz ^{1}H NMR (DMSO-d_6, δ ppm): The spectra showed seven α-protons (δH 4.525, 4.518, 4.446, 4.436, 4.428, 4.177 and 4.168) corresponding to the peptide backbone and long aliphatic chain indicated by CH_2 at 1.55-1.25ppm. 8.72 (one amide proton, 1H and tyrosine –OH, 1H, br), 8.08 (amide proton, 1H, d, j =7.2), 7.73 (amide proton, 1H, br), 7.39-7.34 (amide proton, 1H, br), 7.23-7.15 (amide protons, 2H, br), 7.13-7.03 (amide proton, 1H, br), 7.02-7.00 (amide protons, 2H, br), 6.99-6.93 (amide proton, 3H, m), 6.89-6.86 (tyrosine ring hydrogens, 4H, m); 6.66 (amine proton, 2H, d, j = 8 Hz), 4.52-5.51 (one -OH proton from tyrosine, m), 4.44-4.42 (two -OH protons from serine and threonine and one alpha carbons protons, 2H, m); 4.17-4.16 (two carbons protons, 2H, m); 3.78-3.75 (one α carbon protons, 2H, m); 3.67-3.66 (one α carbon protons, 2H, m); 2.96-2.71 (–CH, 1H, m); 2.59 (-CH 2 2H, m); 2.47-2.45 (-5CH 2 , 10H, m); 2.34 (-CH 2 2H, m); 2.28 (-CH 2 2H, m); 2.17-2.10 (-2CH 2 4H, m); 2.09 (-CH 2 2H, m); 2.04-1.99 (-CH 2 2H, m); 1.89-1.76(-CH 2 2H, m); 1.41(-CH 2 2H, m); 1.24-1.12 (-13CH 2 , 26H, m)and 0.86-0.85 (-CH 3 , 3H, m).

AF$_4$ ^{13}Carbon NMR:

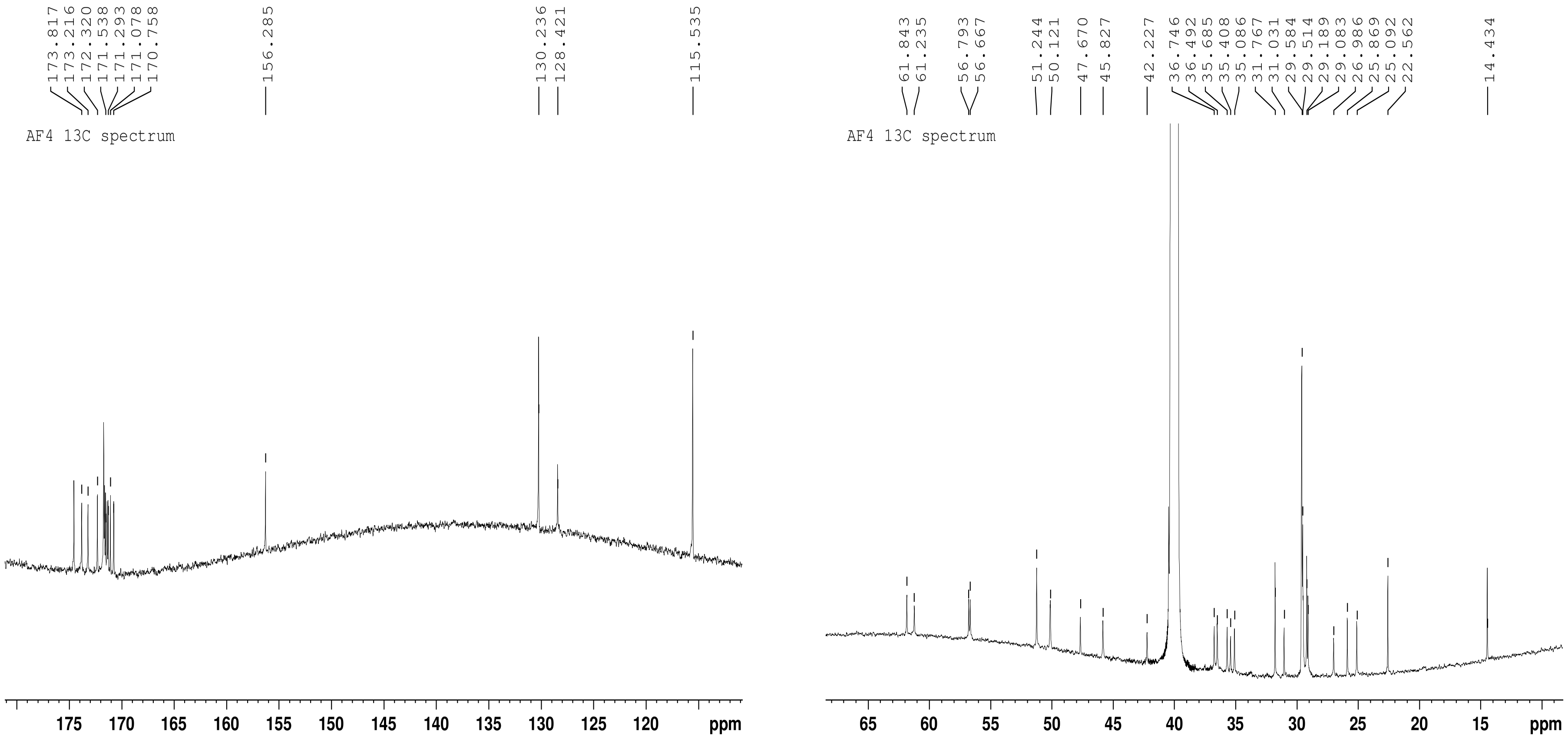

Supplementary Fig. S2.:^{13}C NMR (200 MHz, DMSO-*d*$_6$, δ ppm): The ^{13}C NMR spectrum of the AF$_4$ lipopeptide revealed seven carbonyl carbons in the characteristic range δC 170.75-173.81. Signals at 40.1/40.2 (methylene) and βCH_2 45.827 (methine) are typical to β-amino fatty acid with a long side chain.

AF_4 COSY spectra:

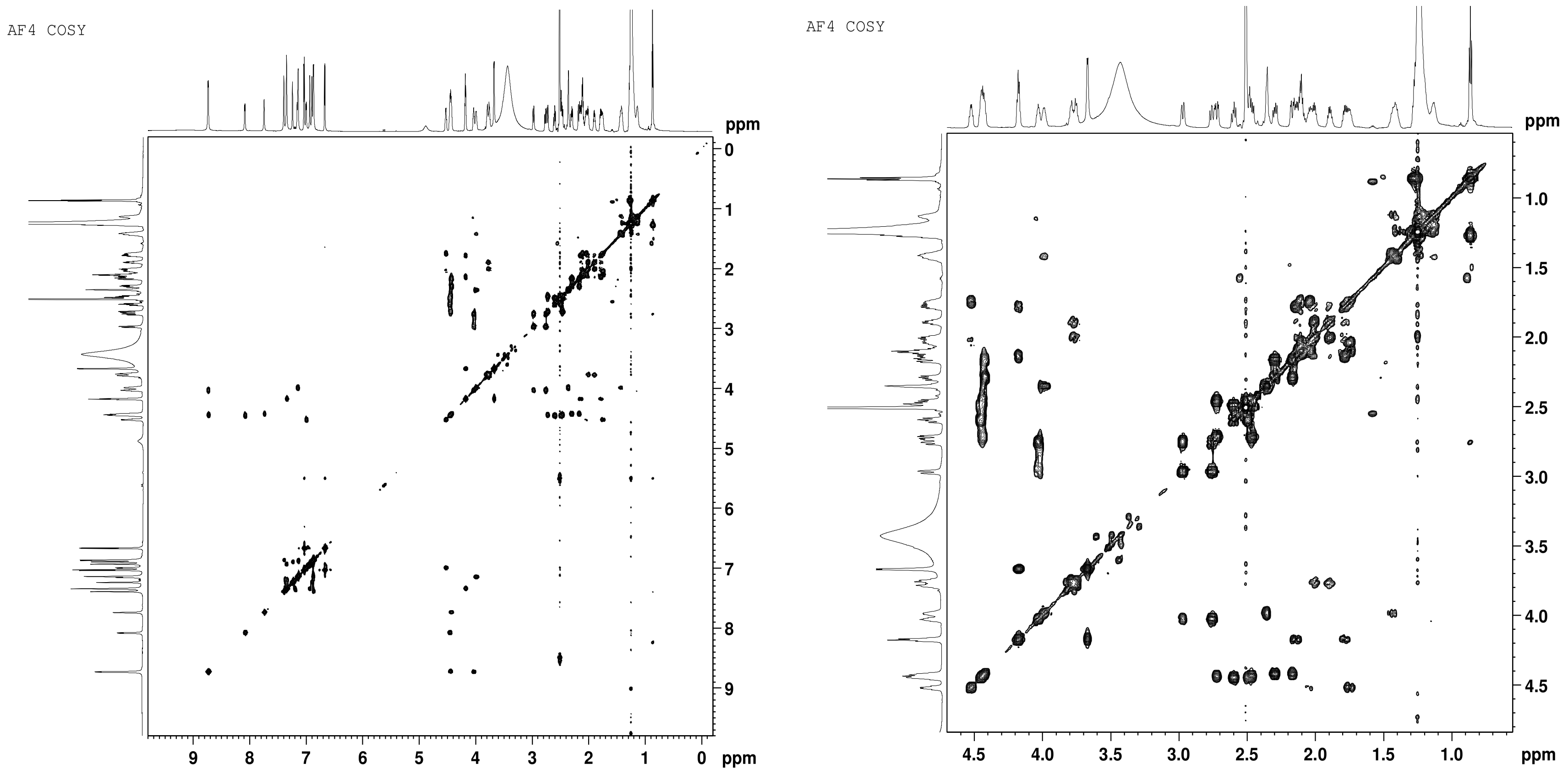

Supplementary Fig. S3.: AF_4 COSY spectra: The presence of a β-amino fatty acid, with the resonance of the β-C proton appearing at δ 4.46 ppm which when potentially coupled with α-C protons of the fatty acid and adjacent protons of the fatty acid chain resulted in resonances at δ 2.0 ppm at δ1.7 ppm

AF$_4$ TOCSY spectra:

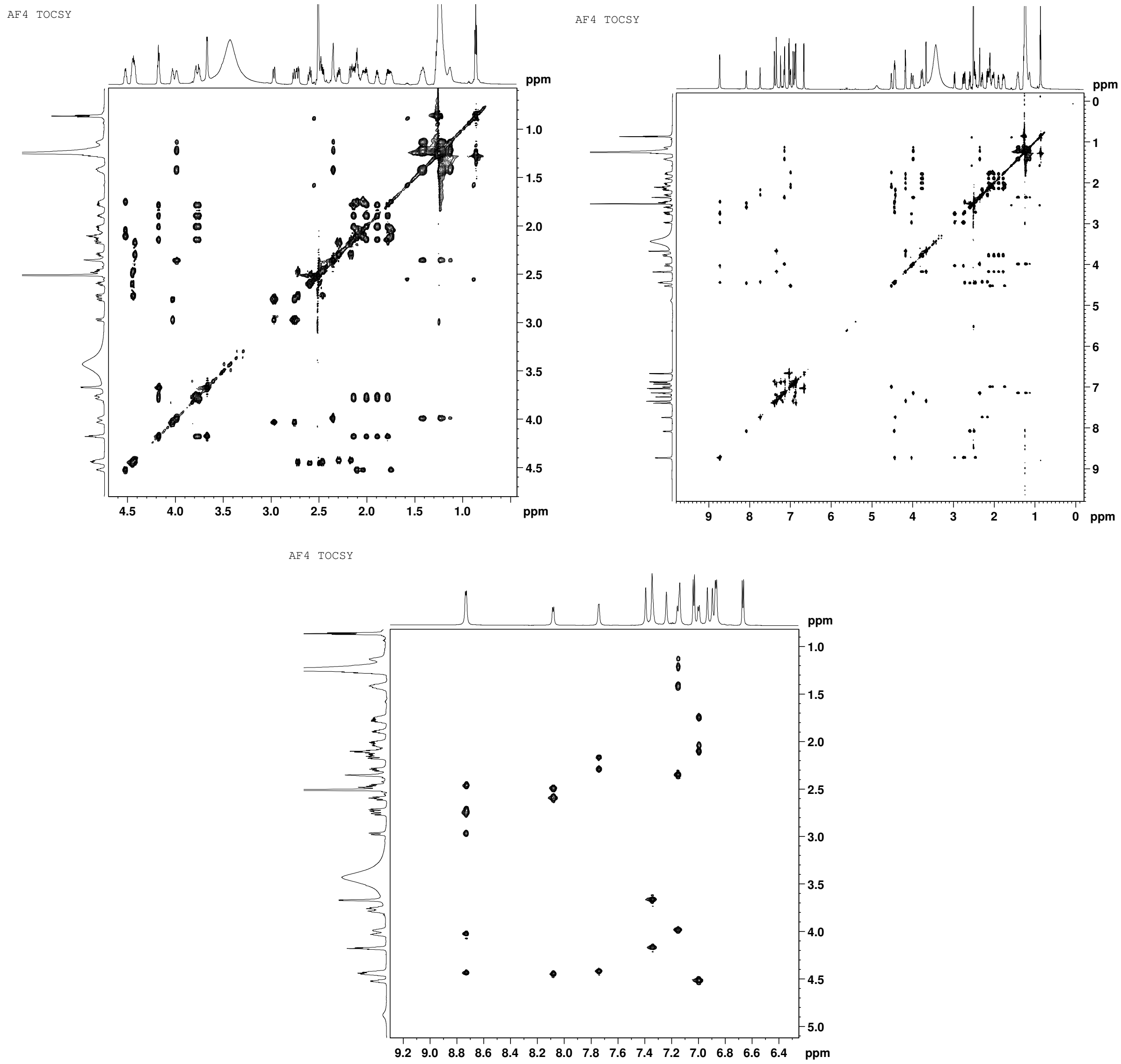

Supplementary Figure S4: AF$_4$ TOCSY spectra: Six spin systems corresponding to 6 amino acids were observed. For Gln and Asn, a TOCSY peak between the chemical shift of 6.9 and 7.6 ppm was observed.
